# Protective influence of betaine on intestinal health by regulating inflammation and improving barrier function in broilers under heat stress

**DOI:** 10.1016/j.psj.2021.101337

**Published:** 2021-06-19

**Authors:** Rashed A. Alhotan, Ali R. Al Sulaiman, Abdulrahman S. Alharthi, Alaeldein M. Abudabos

**Affiliations:** ⁎ Department of Animal Production, College of Food and Agriculture Sciences, King Saud University, P.O. Box 2460, Riyadh 11451, Saudi Arabia; † National Center for Environmental Technology, Life Science and Environment Research Institute, King Abdulaziz City for Science and Technology, P.O. Box 6086, Riyadh 11442, Saudi Arabia

**Keywords:** betaine, heat stress, intestinal inflammation, intestinal barrier function, broiler

## Abstract

This research was executed to study the impacts of adding betaine (**BT**) to broiler diets on intestinal inflammatory response and barrier integrity under heat stress (**HS**). At 21 d of age, 150 male broilers (Ross 308) were randomly assigned to 3 treatment groups: control (**CON**) group, in which broilers were provided standard finisher feed under thermoneutral condition (22 ± 1°C); HS group and HS + BT group, in which broilers were given the standard feed supplied with 0 and 1,000 mg/kg BT, respectively, under cyclic HS condition (33 ± 1°C for 8 h from 08:00 to 16:00 h and the thermoneutral temperature for the residual hours). Each treatment was replicated ten times with 5 broilers per replicate. The HS group showed an elevation (*P* < 0.05) in serum corticosterone (**CORT**) concentration, D-lactate acid (**D-LA**) content, and diamine oxidase (**DAO**) activity, mucosal interleukin-1β (**IL-1β**) level, and expression of heat shock protein 70 (**HSP70**) gene, and a reduction (*P* < 0.05) in mucosal interleukin-10 (**IL-10**) level and secretory immunoglobulin A (**SIgA**) content and relative abundance of mRNA for occludin (**OCLN**), zonula occludens-1 (**ZO-1**), claudin-1 (**CLDN1**), and claudin-4 (**CLDN4**). In contrast, broilers in the HS + BT group exhibited a raise (*P* < 0.05) in mucosal IL-10 level and SIgA content and relative expression of OCLN and ZO-1 genes, and a decline (*P* < 0.05) in serum CORT concentration and DAO activity, mucosal IL-1β level, and expression of HSP70 mRNA. These results indicate that supplemental BT can ameliorate intestinal injury in heat-challenged broilers by suppressing inflammatory responses and enhancing mucosal barrier function.

## INTRODUCTION

The intestinal epithelium plays a pivotal part in the digestion and absorption of nutrients and keeps the bowel's structural barrier integrity (Nawab et al., 2018). Hence, the intactness of the epithelial barrier is crucial for broilers’ wellness and productivity. The barrier is composed of a monocular stratum of epithelial cells, tightly linked with each other by intercellular junctional complexes, including adherens junctions (**AJ**) and tight junctions (**TJ**). It forms a distinguished network, controlling the permeability of intestinal epithelium and safeguarding mucosal tissues from deleterious substances existent within the luminal environment (Lechuga and Ivanov, 2017). Nevertheless, it has been confirmed that the overproduction of reactive species and proinflammatory cytokines during exposure to heat stress (**HS**) would downregulate the apical junctional complexes, giving rise to weakened intestinal barrier function (Lian et al., 2020). Dysfunction of this barrier elevates enteric permeability to endotoxins, contributing to the local and systemic inflammation and immune injuries (Alhenaky et al., 2017).

Dietary strategies have been demonstrated to ameliorate the influence of HS on animals by lowering the adverse consequences of gut impairment, inclusive of plant-derived products such as betaine (**BT**) which is synthesized as a by-product of beetroots processing (Saeed et al., 2017). Research reports have informed that BT performs various biologic activities within tissues, comprising antistress (Ratriyanto and Mosenthin, 2018), osmotic regulation (Figueroa-Soto and Valenzuela-Soto, 2018), methyl donor (Zou et al., 2016), antioxidant (Adjoumani et al., 2017), and anti-inflammatory (Yang et al., 2018). Recent studies showed that BT supplementation could stimulate antioxidant defenses (Akhavan-Salamat and Ghasemi, 2016), enhance immune response (Ghasemi and Nari, 2020), as well as ameliorate intestinal barrier functions (Shakeri et al., 2019) in broilers undergone HS conditions. Nevertheless, there are minimal research reports regarding the impacts of BT on high temperature-induced intestinal damage in broilers, particularly on the inflammatory response, mucosal immune, and mucosal barrier gene expression. Only Liu et al. (2020) reported that dietary BT enhanced barrier function-associated gene expression levels of the small intestinal mucosa in indigenous broilers exposed to a long-term HS environment. Based on the advantageous impacts of BT, it has been hypothesized that the impairment of anti-inflammatory and barrier activities in broilers’ intestinal mucosa caused by HS may be ameliorated by BT supplementation. Consequently, this research was performed to assess the possible protective impacts of BT supplement upon corticosterone (**CORT**) hormone and intestinal permeability, inflammatory reaction, immunity, plus expression patterns of heat shock protein 70 (***HSP70***), AJ, and TJ genes in broilers submitted to cyclic HS.

## MATERIALS AND METHODS

### Experimental Design and Husbandry

All proceedings concerning birds in the current research were approved by the Research Ethics Committee of King Saud University, Riyadh, Saudi Arabia (KSU‐SE‐20‐22).

One hundred fifty 21-day-old male broilers (Ross 308) with comparable body weight were randomly divided into 3 treatment groups, each of which comprised 10 replicates with 5 chickens per replicate. The first group was the control (**CON**), in which chickens were provided a standard finisher diet under thermoneutral condition (22 ± 1°C); the second and third groups were HS and HS + BT, in which chickens were fed the standard diet supplemented with 0 and 1,000 mg/kg BT, respectively, under cyclic HS condition (33 ± 1°C for 8 h from 08:00 to 16:00 and 22 ± 1°C for the rest time per d). The supplemental level of BT (Betafin, 97% natural BT, Danisco Animal Nutrition, Marlborough, Wilts, UK) was chosen in accordance with a previous study in indigenous chickens reared under long-lasting cyclic HS (Liu et al., 2020).

Broilers were housed in battery cages (125 cm × 65 cm × 50 cm, L × W × H) in 2 environmentally controlled rooms. The treatments lasted for 21 consecutive d, during which the temperature was continuously recorded in each room using Data Loggers (Part # UX100-011A, HOBO, Onset Computer Corporation, Bourne, MA). A 50 to 60% relative humidity and a 23L: 1D lighting program were maintained throughout the trial period. The birds were monitored several times during the trial period on a daily basis, especially those under HS, and suitable practices were taken accordingly. A diet based on corn and soybean meal (Table 1) was prepared to fulfill the nutritional specifications of broilers as suggested by Ross broiler's guide (Aviagen, 2019). Chickens had free access to mash feed and water throughout the trial period.

**Table 1:** Ingredients and dietary composition (as fed) of the standard finisher diet.

Ingredients	Amount, %
Yellow corn	60.16
Soybean meal	26.41
Corn gluten meal	5.73
Corn oil	3.90
Di-calcium phosphate	1.56
Limestone	1.04
L-Lysine HCL	0.33
DL-Methionine	0.11
L-Threonine	0.09
Sodium chloride	0.34
Vitamin & Mineral premix^1^	0.35
Calculated analysis^2^ (%, except for ME)	
ME, kcal/kg	3,200
CP	20
Ca	0.81
Available P	0.41
Dig. Lys	1.06
Dig. TSAA	0.83
Dig. Thr	0.71

1 Provides per kg diet: vitamin A, 2400000 IU; vitamin D, 1000000 IU; vitamin E, 16000 IU; vitamin K, 800 mg; vitamin B1, 600 mg; vitamin B2, 1600 mg; vitamin B3, 8000 mg; vitamin B5, 3000 mg; vitamin B6, 1000 mg; vitamin B7, 40 mg; vitamin B9, 400 mg; vitamin B12, 6 mg; antioxidants, 3000 mg; Co, 80 mg; Cu, 2000 mg; I, 400; Fe, 1200 mg; Mn, 18000 mg; Se, 60 mg, and Zn, 14000 mg.

2 Calculated based on AMINODAT 5.0 (Evonik Animal Nutrition, Hanau-Wolfgang, Germany).

### Sample Collection

At the end of the experimental period (42 d), one bird from each replicate was randomly chosen for specimen collection. Blood samples were obtained from the brachial vein and centrifuged at 2,000 × *g* for 10 min in a refrigerated centrifuge to segregate sera, which were afterward stored at −80°C for the analysis of related parameters.

After slaughtering, the jejunum was gathered and emptied utilizing gentle pressure. Approximately 10-cm long sections from the middle of the jejunum were cut, opened longitudinally, and rinsed gently with chilled phosphate-buffered saline for collecting mucosa. The mucosa from each specimen was gathered utilizing a sterile microscope slide, quickly kept in liquid nitrogen, and afterward stored at −80°C for the examination of immunologic parameters and mRNA expression.

### Measurement of Serum Indices

The serum CORT concentration (catalog No. MBS754020), D-lactate acid content (**D-LA**, catalog No. MBS754371), and diamine oxidase activity (**DAO**, catalog No. MBS743254) were assayed spectrophotometrically using ELISA kits (MyBioSource Inc., San Diego, CA) as reported by the manufacturer's protocols.

### Measurement of Intestinal Immune Parameters

The jejunal mucosa samples were diluted and homogenized with chilled phosphate-buffered saline employing Ultra-Turrax homogenizer (Model T25, IKA Works, Inc., Wilmington, NC). The homogenates were centrifuged at 4,450 × *g* for 15 min in a refrigerated centrifuge to collect the supernatants, which were immediately stored at −20°C until analysis. The levels of total protein (catalog No. MBS165636), interleukin-1β (**IL-1β**, catalog No. MBS761055), interleukin-10 (**IL-10**, catalog No. MBS701683), and secretory immunoglobulin A (**SIgA**, catalog No. MBS737239) in the supernatant were analyzed using ELISA kits (MyBioSource Inc., San Diego, CA). All methods were conducted as reported by the manufacturer's protocols. The outcomes were normalized against total protein contents for comparison between samples.

### Quantification of Messenger RNA

The total RNA of the jejunal tissue samples was insulated employing TRIzol Reagent (Catalog No. 15596026, Thermo Fisher Scientific, Waltham, MA) as reported by the manufacturer's protocol. The integrity of isolated RNA was examined by agarose gel electrophoresis, while its concentration and purity were quantified using a Nanodrop Spectrophotometer (ND-2000, Thermo Fisher Scientific). Total RNA samples were then quantitatively converted to cDNA utilizing High-Capacity cDNA Reverse Transcription Kit (Catalog No. 4368814, Thermo Fisher Scientific) as reported by the manufacturer's protocol.

Real-time PCR was carried out using the StepOnePlus Real-Time PCR System (Catalog NO. 4376600, Thermo Fisher Scientific) in a reaction mixture consisted of 2 μL of cDNA sample, 0.4 μL of forward primer, 0.4 μL of reverse primer, 10 μL of Taq DNA Polymerase (Catalog No. 10342020, Thermo Fisher Scientific), 0.4 μL of ROX Reference Dye (Catalog No. 12223012, Thermo Fisher Scientific), and 6.8 μL of double-distilled water. The sequences of forward and reverse primers for the designated and referential genes are shown in Table 2. The PCR process comprised an initial run at 95°C for 30 s, 40 cycles of denaturation at 95°C for 5 s, and an annealing step at 60°C for 30 s. Following amplification, melting curve analysis was implemented to validate the specificity and purity of each PCR-amplified product under the following conditions, one cycle of denaturation at 95°C for 10 s, followed by a rise in temperature from 65 to 95°C at a rate of 0.5°C/s. The fold change expression of the designated genes (*HSP70*, E-cadherin, occludin (***OCLN***), zonula occludens-1 (***ZO-1***), claudin-1 (***CLDN1***), and claudin-4 (***CLDN4***)) normalized to β-actin and relative to the CON group was quantified employing the formula 2^−ΔΔCT^ as previously described by Livak and Schmittgen (2001).

**Table 2:** Sequences of real-time PCR primers.

Genes^1^	Accession number	Primer sequence (5′→3′)	Product size (bp)
HSP70	NM_001006685.1	Forward: CGTCAGTGCTGTGGACAAGAGTA	144
		Reverse: CCTATCTCTGTTGGCTTCATCCT	
E-cadherin	NM_001039258.2	Forward: CAGAAGAGGGATTGGGTCAT	248
		Reverse: GCGTGGGATAAGAGGGTGTA	
OCLN	NM_205128.1	Forward: CCGTAACCCCGAGTTGGAT	214
		Reverse: ATTGAGGCGGTCGTTGATG	
ZO-1	XM_413773.4	Forward: TGTAGCCACAGCAAGAGGTG	159
		Reverse: CTGGAATGGCTCCTTGTGGT	
CLDN1	NM_001013611	Forward: GGTGAAGAAGATGCGGATGG	139
		Reverse: TCTGGTGTTAACGGGTGTGA	
CLDN4	AY435420	Forward: ATCGCCCTGTCCGTCATC	137
		Reverse: ACCACGCAGTTCATCCACAG	
β-actin	NM_205518.1	Forward: TTGGTTTGTCAAGCAAGCGG	100
		Reverse: CCCCCACATACTGGCACTTT	

1 CLDN1, claudin-1; CLDN4, claudin-4; HSP70, Heat Shock Protein 70; OCLN, occludin; ZO-1, zonula occludens-1.

### Statistical Analysis

Data were analyzed based on one-way ANOVA employing the GLM procedure of SAS software (SAS Institute Inc., version 9.4, Cary, NC). Differences between means were separated by Tukey's test at a significance level of *P* < 0.05. Figures were drawn utilizing GraphPad Prism (GraphPad software Inc., version 9.0. 2, San Diego, CA). Findings are exhibited as mean + SEM.

## RESULTS

### Serum CORT Concentration

**Figure 1:**
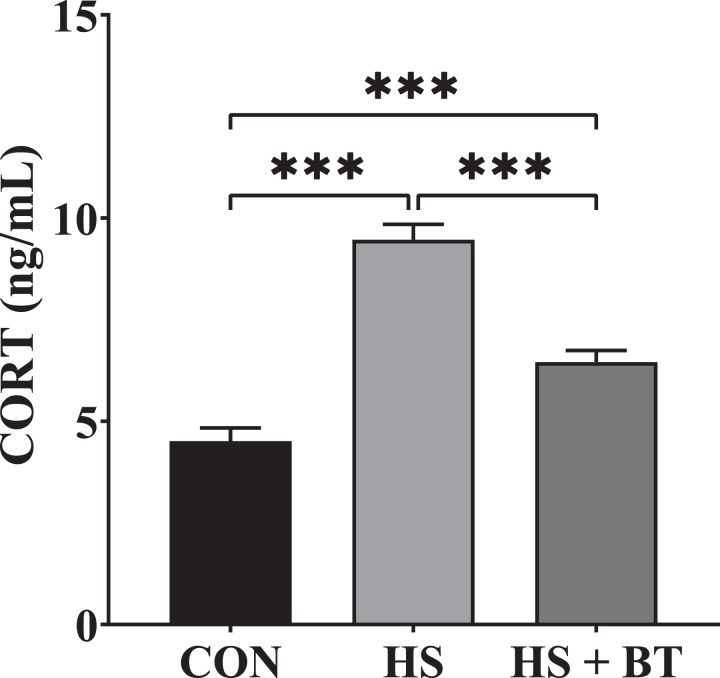
Corticosterone (CORT) concentration in the serum of broilers fed supplemental betaine (BT) under cyclic heat stress (HS) at 42 d. Treatments: control (CON), standard diet + thermoneutral temperature (22 ± 1°C); HS, standard diet + cyclic HS (33 ± 1°C for 8 h. and 22 ± 1°C for 16 h/d); HS + BT, 1,000 mg BT/kg standard diet + cyclic HS as the HS group. The values are expressed as mean + SEM (n = 10). ***P ≤ 0.001.

As shown in Figure 1, at 42 d, birds in the HS group manifested an elevation in CORT concentration compared to those in the CON group (*P* < 0.05). Whereas birds in the HS + BT group demonstrated a reduction in CORT concentration compared to those in the HS group (*P* < 0.05).

### Serum D-LA Content and DAO Activity

**Figure 2:**
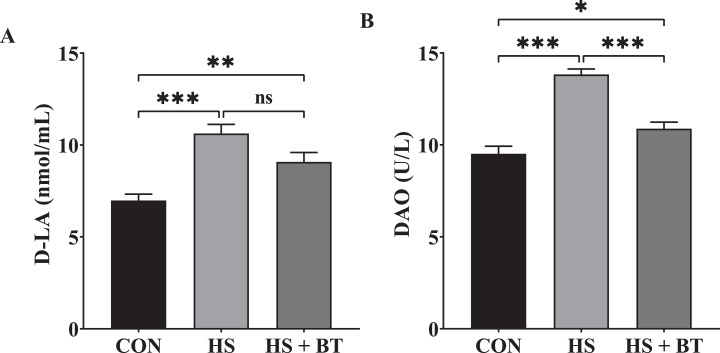
D-lactate acid (D-LA) content (A) and diamine oxidase (DAO) activity (B) in the serum of broilers fed supplemental betaine (BT) under cyclic heat stress (HS) at 42 d. Treatments: control (CON), standard diet + thermoneutral temperature (22 ± 1°C); HS, standard diet + cyclic HS (33 ± 1°C for 8 h and 22 ± 1°C for 16 h/d); HS + BT, 1,000 mg BT/kg standard diet + cyclic HS as the HS group. The values are expressed as mean + SEM (n = 10). *P ≤ 0.05, **P ≤ 0.01, ***P ≤ 0.001.

As shown in Figure 2, at 42 d, birds in the HS group manifested an elevation in D-LA content and DAO activity compared to those in the CON group (*P* < 0.05). Whereas birds in the HS + BT group demonstrated a reduction in DAO activity compared to those in the HS group (*P* < 0.05).

### Jejunal Mucosal Interleukin Levels

**Figure 3:**
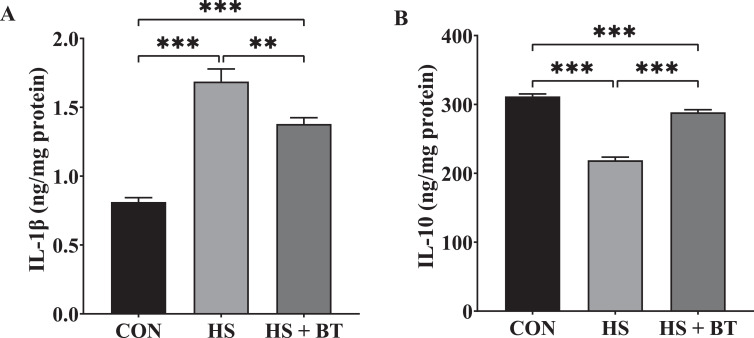
Interleukin-1β (IL-1β) level (A) and interleukin-10 (IL-10) level (B) in the jejunal mucosa of broilers fed supplemental betaine (BT) under cyclic heat stress (HS) at 42 d. Treatments: control (CON), standard diet + thermoneutral temperature (22 ± 1°C); HS, standard diet + cyclic HS (33 ± 1°C for 8 h and 22 ± 1°C for 16 h/d); HS + BT, 1,000 mg BT/kg standard diet + cyclic HS as the HS group. The values are expressed as mean + SEM (n = 10). **P ≤ 0.01, ***P ≤ 0.001.

As shown in Figure 3, at 42 d, birds in the HS group manifested an elevation in IL-1β level and a reduction in IL-10 level compared to those in the CON group (*P* < 0.05). Whereas birds in the HS + BT group demonstrated a reduction in IL-1β level and an elevation in IL-10 level compared to those in the HS group (*P* < 0.05).

### Jejunal Mucosal SIgA Content

**Figure 4:**
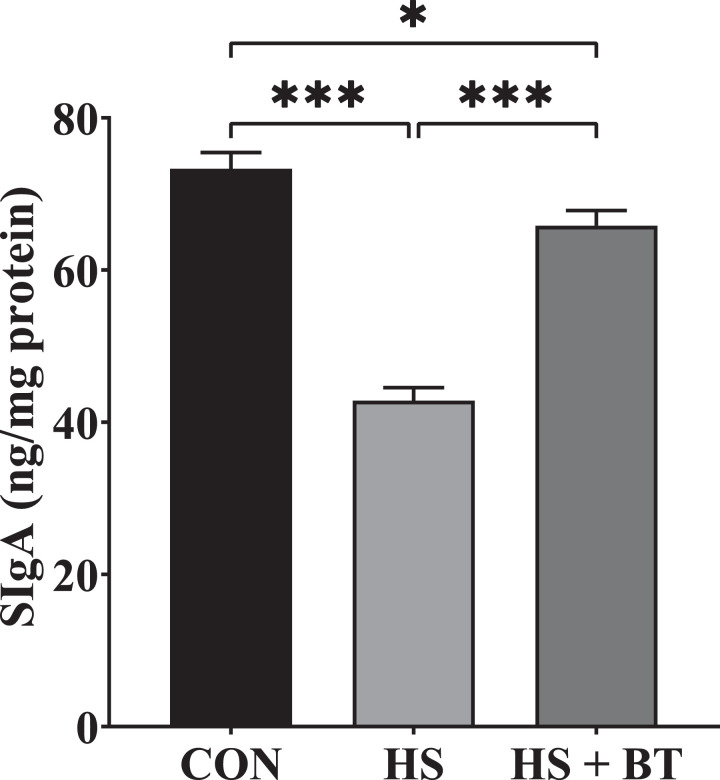
Secretory Immunoglobulin A (SIgA) content in the jejunal mucosa of broilers fed supplemental betaine (BT) under cyclic heat stress (HS) at 42 d. Treatments: control (CON), standard diet + thermoneutral temperature (22 ± 1°C); HS, standard diet + cyclic HS (33 ± 1°C for 8 h and 22 ± 1°C for 16 h/d); HS + BT, 1,000 mg BT/kg standard diet + cyclic HS as the HS group. The values are expressed as mean + SEM (n = 10). *P ≤ 0.05, ***P ≤ 0.001.

As shown in Figure 4, at 42 d, birds in the HS group manifested a reduction in SIgA content compared to those in the CON group (*P* < 0.05). Whereas birds in the HS + BT group demonstrated an elevation in SIgA content compared to those in the HS group (*P* < 0.05).

### Jejunal Mucosal HSP70 Gene Expression

**Figure 5:**
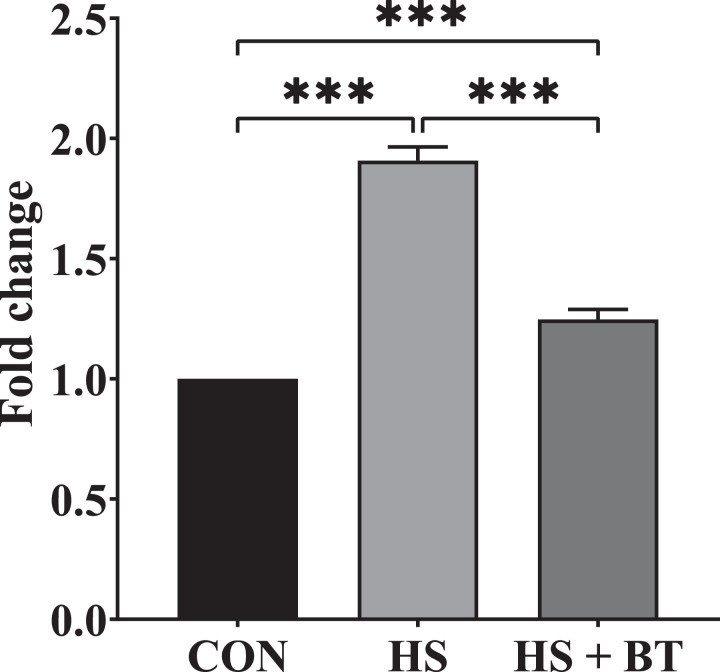
Relative mRNA expression of heat shock protein 70 (HSP70) gene in the jejunal mucosa of broilers fed supplemental betaine (BT) under cyclic heat stress (HS) at 42 d. Treatments: control (CON), standard diet + thermoneutral temperature (22 ± 1°C); HS, standard diet + cyclic HS (33 ± 1°C for 8 h and 22 ± 1°C for 16 h/d); HS + BT, 1,000 mg BT/kg standard diet + cyclic HS as the HS group. The values are expressed as mean in arbitrary unit + SEM (n = 10). The mRNA level for the CON group was used as a calibrator. ***P ≤ 0.001.

As shown in Figure 5, at 42 d, birds in the HS group manifested an upregulation in *HSP70* mRNA expression compared to those in the CON group (*P* < 0.05). Whereas birds in the HS + BT group demonstrated a downregulation in *HSP70* mRNA expression compared to those in the HS group (*P* < 0.05).

### Jejunal Mucosal AJ and TJ Gene Expression

**Figure 6:**
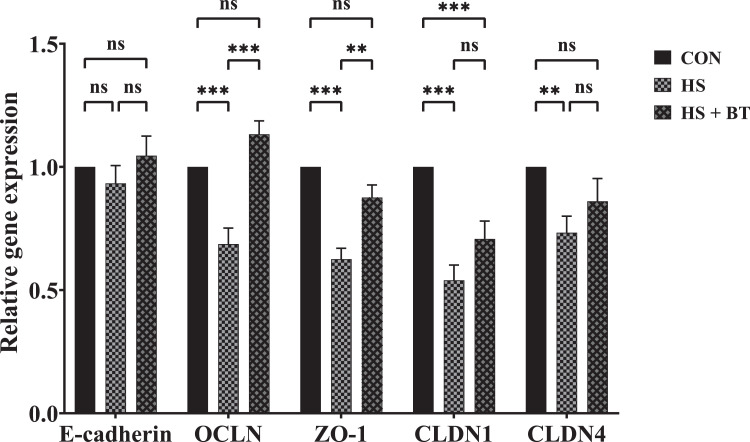
Relative mRNA expression of genes related to jejunal adherens and tight junctions of broilers fed supplemental betaine (BT) under cyclic heat stress (HS) at 42 d. Treatments: control (CON), standard diet + thermoneutral temperature (22 ± 1°C); HS, standard diet + cyclic HS (33 ± 1°C for 8 h and 22 ± 1°C for 16 h/d); HS + BT, 1,000 mg BT/kg standard diet + cyclic HS as the HS group. The values are expressed as mean in arbitrary unit + SEM (n = 10). The mRNA level for the CON group was used as a calibrator. **P ≤ 0.01, ***P ≤ 0.001. Abbreviations: CLDN1, claudin-1; CLDN4, claudin-4; OCLN, occludin; ZO-1, zonula occludens-1.

As shown in Figure 6, at 42 d, birds in the HS group manifested a down-regulation in mRNA expression levels of *OCLN, ZO-1, CLDN1*, and *CLDN4* compared to those in the CON group (*P* < 0.05). Whereas birds in the HS + BT group demonstrated an up-regulation in mRNA abundance of *OCLN* and *ZO-1* compared to those in the HS group (*P* < 0.05). However, no differences were found in the mRNA level of E-cadherin among the treatments (*P* > 0.05).

## DISCUSSION

Heat-stressed animals experience various pathophysiological changes. Former studies on broilers have reported that HS can raise serum CORT concentration which can be regarded as a credible index of stress (Quinteiro-Filho et al., 2017; Cheng et al., 2018). In the current research, cyclic HS predictably raised serum CORT values, and this surge in circulating CORT was relieved by BT supplementation. This finding implies that natural BT could alleviate the stress resulting from heat and its adverse impacts on broilers to some extent, which was supported by enhancing the health status of the HS + BT group. The result reported herein is in line with the data of Chen et al. (2020), who showed that supplemental BT could reduce the concentration of CORT in the sera of broilers subjected to transport stress. This positive influence may be due to the activity of BT as a methyl donor through reducing the presence of homocysteine in the brain during the methionine cycle (Olthof and Verhoef, 2005).

Maintaining the functionality of the intestinal barrier is considerable to bird health and livability. When any damage to the intestinal mucosa occurs, the permeability of the intestinal barrier raises, allowing significant quantities of D-LA, the end-product of intraintestinal bacteria, and DAO, an endocellular enzyme existing in the intestinal villi, to pass into the bloodstream (Chen et al., 2017). Thus, blood levels of D-LA and DAO could be utilized as dependable indicators that measuring the extent of mucosal barrier injury. In the current research, cyclic HS raised D-LA contents and DAO activities in the sera, which is consonant with the analyses of Liu et al. (2018) and Cheng et al. (2019), who observed a considerable surge in the release of D-LA and DAO into broilers' blood during heat exposure. Recently, Wang et al. (2020) found that piglet diets supplemented with BT markedly reduced DAO activity in the plasma under normal conditions. Similarly, in the current research, dietary inclusion of BT declined serum DAO activity of birds exposed to HS, suggesting that BT exerts protective influences to some extent against HS-induced intestinal barrier dysfunction.

Additionally, the HS group showed a significant increment in the generation of IL-1β with a reduction in the liberation of IL-10 in the jejunal mucosa of broilers; these findings are consonant with a former study on heat-challenged broilers (Song et al., 2017). However, this imbalance in the inflammatory reaction was significantly regulated in the HS + BT group. In a similar way, Shi et al. (2019) observed that BT treatment in vitro markedly diminished the formation of proinflammatory cytokines (Tumor Necrosis Factor Alpha, IL-6, and IL-1β) and augmented the creation of anti-inflammatory cytokine (**IL-10**) in N9 microglial cells treated with lipopolysaccharide in a dose-dependent manner. It has been established that exaggerated generation of nitric oxide (**NO**) radical during heat exposure provokes the activation of inflammatory signaling cascades, which stimulates proinflammatory cytokines through animating the transcription factor kappa-B. Inflammatory cells liberate several reactive species at the injury site, bringing on magnified oxidative stress and intestinal tissue damage (Saracila et al., 2021). Conversely, BT has been shown to prevent NO generation markedly by inhibiting the expression of NO synthase, a principal microglial enzyme for NO formation, consequently repressing inflammation in the intestinal wall (Zhao et al., 2018). Moreover, the in vivo results of Tang et al. (2021) manifested that HS-caused inflammation in the intestinal epithelium was correlated with the overexpression of histone deacetylases, which plays a significant role in maintaining proinflammatory mediators' production by controlling the intracellular signaling pathway of Toll-like receptor 4/nuclear factor-kappa B. On the other hand, BT was found to inhibit inflammatory response possibly by regulating the Toll-like receptor 4/nuclear factor-kappa B signaling pathway and histone deacetylases expression to repair damaged tissue (Li et al., 2015). Additional data is required to illustrate the mechanisms fully.

The equilibrium among pro- and anti-Inflammatory cytokines is a required factor for gut immunological homeostasis (Yue et al., 2017). Secretory IgA is an antibody that acts as the first line of defending the intestinal lumen from antigens and pathogens by preventing their access to epithelial cell receptors (Mantis et al., 2011). Song et al. (2018) observed that broilers challenged with HS manifested a reduction in SIgA and IgG contents in the jejunal mucosa. Likewise, the present research demonstrated that the content of SIgA in the jejunum mucosa was reduced by HS treatment, which could be partly related to elevated CORT release because of changes in the function of a hypothalamic–pituitary–adrenal axis. Sun et al. (2020) showed that supplemented low Met diet with BT enhanced the intestinal immunity of on-growing grass carp. Similarly, in this study, the jejunal SIgA content of heat-challenged birds was markedly ameliorated by dietary BT, which may be associated with its role in reducing excess production of proinflammatory cytokines following HS to keep a regulated cytokine environment.

It is well known that the overexpression of *HSP70* in response to either environmental or physiological stress induces protein folding to prevent protein aggregation and subsequent cell death by free radicals through its influence on mitochondrial permeability (Powers et al., 2010). Evidence is growing that the HSP70 interacts with inflammatory cytokines to diminish inflammation by boosting the immunity of the small intestine (Borges et al., 2012). In the present research, there was overexpression in *HSP70* mRNA under HS conditions which is consonant with the former studies (Vesco et al., 2020; Siddiqui et al., 2020). Comparable to Li et al. (2019) study, BT treatment alleviated heat-induced increased *HSP70* mRNA expression. The downregulation of *HSP70* expression in the HS + BT group could be related to the stabilization of intestinal epithelial cell proteins due to the osmotic properties of BT (Bruździak et al., 2013).

The AJ, including transmembrane spanning and adhesive receptors, and TJ, including transmembrane and peripheral membrane proteins, are the main constituents of the enteric mucosal barrier that are mainly accountable for controlling paracellular permeability and regulating barrier activity (Campbell et al., 2017). Therefore, the dysfunction of these multiple junctional complexes is harmful to the barrier function. However, Zhang et al. (2017) found that HS markedly declined the relative abundances of *CLDN1, OCLN, ZO-1*, and E-cadherin in the jejunum of broilers. Consistently, this research showed that expression levels of *OCLN, ZO-1, CLDN1*, and *CLDN4* in the jejunum mucosa were reduced by HS, which are possibly accountable for the elevation of intestinal permeability. A potential explication for HS-induced injury in the intestinal mucous membrane is that hyperthermia and the subsequent intestinal ischemia and hypoxia lead to an overproduction of reactive species, causing epithelial shedding (Yu et al., 2013). This acute loss of epithelial cells is almost always accompanied by elevated intestinal permeability to endotoxins, which could also explicate the incidence of mucosal inflammation in the HS birds. It has also been informed that inflammation-associated intestinal oxidative stress could induce barrier impairment by reducing the expression of TJ-related protein genes (Alhenaky et al., 2017). Betaine has been formerly reported to perform various cellular activities involving antioxidant, osmoregulation, and methyl-donor (Figueroa-Soto and Valenzuela-Soto, 2018). Nevertheless, the precise molecular mechanisms concerning how BT promotes the intestinal barrier function are still not clear. In the current study, similar to Liu et al. (2020), broilers fed a BT-supplemented diet under HS exhibited a significant upregulation in the jejunal gene expressions of *OCLN* and *ZO-1*. In line with these findings, Wu et al. (2020) found that BT could alleviate the downregulation of TJ proteins triggered by lipopolysaccharide in the intestinal porcine epithelial cells. Although the application of dietary BT on mitigating HS has been widely carried out in poultry, studies involving the protecting influence of dietary BT against HS-caused downregulation of enteric TJ or AJ proteins are extremely limited; accordingly, no further comparisons could be executed. In terms of the mode of action, BT participated in the osmoregulation of the enteric epithelium and had a favorable impact on water locomotion over the epithelial cells in vitro (Wu et al., 2020). Also, it was assumed that the affirmative influence of BT on the expression of TJ-related genes in heat-exposed broiler birds could be attributed to its antioxidative characteristics through the homocysteine-methionine cycle. The BT exhibited the capability to diminish the formation of reactive oxygen species in broilers under an elevated-temperature environment (Shakeri et al., 2018), demonstrating that betaine could be relieving oxidative damage-triggered intestinal tissue injury. However, the specific mechanisms need additional investigations.

In conclusion, the current research findings manifested that BT supplementation can partly safeguard the intestinal health of broiler bird against HS by regulating the cytokine formation and upregulating the expression of TJ-associated genes. These improvements could be attributed to its transmethylation and osmoregulatory activities that assist in safeguarding intestinal epithelial cells and so limiting performance losses during HS.
